# National Kidney Month — March 2018

**DOI:** 10.15585/mmwr.mm6710a1

**Published:** 2018-03-16

Each year, March is designated National Kidney Month to raise awareness about the prevention and early detection of kidney disease. In the United States, kidney diseases are the ninth leading cause of death (*1*). Among U.S. adults aged ≥20 years, 15% (30 million persons) are estimated to have chronic kidney disease. Chronic kidney disease is defined as damaged kidneys or a glomerular filtration rate (i.e., a measure of kidney function) <60 mL/min/1.73 m^2^ for more than 3 months (*2*,*3*). Chronic kidney disease is also estimated to be more common in women than in men (*2*,*3*). However, among persons with moderate to severe chronic kidney disease, awareness of having the disease was lower in women than in men (*3*). Risk factors for chronic kidney disease include diabetes, high blood pressure, cardiovascular disease, and obesity (*2*); controlling diabetes and high blood pressure can delay or prevent chronic kidney disease and improve health outcomes (*2*). CDC supports the Chronic Kidney Disease Surveillance System (https://www.cdc.gov/ckd/surveillance) to document and monitor kidney disease and its risk factors in the U.S. population and to track progress in kidney disease prevention, detection, and management. This week's *MMWR* issue includes a report on acute kidney injury, a risk factor for developing or worsening chronic kidney disease. Information is available about kidney disease prevention and control at https://www.nkdep.nih.gov/ and about diabetes prevention and control at https://www.cdc.gov/diabetes.
